# Monodisperse 130 kDa and 260 kDa Recombinant Human Hemoglobin Polymers as Scaffolds for Protein Engineering of Hemoglobin-Based Oxygen Carriers

**DOI:** 10.3390/jfb3010061

**Published:** 2012-01-13

**Authors:** David A. Marquardt, Michael P. Doyle, Jeffrey S. Davidson, Janet K. Epp, Jacqueline F. Aitken, Douglas D. Lemon, Spencer J. Anthony-Cahill

**Affiliations:** 1 Research and Development, Baxter Hemoglobin Therapeutics, 2545 Central Avenue, Boulder, CO 80301, USA; E-Mails: marquardt_david_a@lilly.com (D.A.M.); mdoyle@clovisoncology.com (M.P.D.); douglemon@yahoo.com (D.D.L.); 2 Somatogen Inc., 2545 Central Avenue, Boulder, CO 80301, USA; E-Mails: info@pisces-molecular.com (J.K.E.); j.aitken@auckland.ac.nz (J.F.A.)

**Keywords:** polyhemoglobin, dihemoglobin, tetrahemoglobin, hemoglobin engineering, extravasation, pressor response

## Abstract

A recombinant 130 kDa dihemoglobin which is made up of a single-chain tetra-α globin and four β globins has been expressed as a soluble protein in *E. coli*. The sequence of the single chain tetra-α is: αI-Gly-αII-(SerGlyGly)5Ser-αIII-Gly-αIV. This dihemoglobin has been purified and characterized *in vitro* by size exclusion chromatography, electrospray mass spectroscopy, equilibrium oxygen binding, and analytical ultracentrifugation. The observed values of P_50_ and n_max_ for the dihemoglobin are slightly lower than those observed for the recombinant hemoglobin rHb1.1 (a “monohemoglobin” comprised of two β globins and an αI-Gly-αII diα-globin chain). Titration of the deoxy form of dihemoglobin with CO shows that all eight heme centers bind ligand**.** *In vivo*, dihemoglobin showed increased circulating halflife and a reduced pressor response in conscious rats when compared to rHb1.1. These observations suggest that dihemoglobin is an oxygen carrying molecule with desirable *in vivo* properties and provides a platform for an isooncotic hemoglobin solution derived solely from a recombinant source. A 260 kDa tetrahemoglobin has also been produced by chemical crosslinking of a dihemoglobin that contains a Lys16Cys mutation in the C-terminal α-globin subunit. Tetrahemoglobin also shows reduced vasoactivity in conscious rats that is comparable to that observed for dihemoglobin.

## 1. Introduction

Hemoglobin-based oxygen carriers have been studied for many years as potential therapeutics [1,2,3,4,5]. The hemoglobins required to produce candidate therapeutics have been obtained from outdated human blood, transgenic and other animal sources, and bacterial or yeast fermentation. Solutions of unmodified cell-free hemoglobin (Hb) exhibit renal toxicity due to dissociation of α_2_β_2_ tetramers into αβ dimers (which are small enough to be filtered in the kidneys). This toxicity can be prevented by covalent linking of the globin subunits using a variety of chemical crosslinking agents, or by gene fusion (reviewed in [1,2,3,4,5]). Production of hemoglobin by recombinant DNA technology provides the ability to selectively alter the hemoglobin structure and function by genetic manipulation, and many point mutations which affect hemoglobin function have been identified and characterized [6,7]. For example, replacement of Asn108 with Lys in the β globins and covalent linkage of the two α globins in the human α_2_β_2_ tetramer by insertion of a single Gly codon between two α genes yields a hemoglobin, rHb1.1, which binds oxygen in a cooperative manner with a P_50_ close to that of human blood [8]. This example demonstrates that a relatively simple structural alteration (*i.e*., linking the α globins) can yield a protein with improved *in vivo* function.

Preclinical and clinical experiences with solutions of hemoglobin-based oxygen carriers (HBOCs) have identified several other severe side-effects, such as pulmonary and systemic vasoactivity, myocardial lesions, elevation of serum enzyme levels, and complement activation [2,3,9]. Many of these side-effects are hypothesized to result from scavenging of nitric oxide (NO) by oxy-Hb following extravasation of the cell-free Hb into extravascular spaces [9,10]. NO scavenging activity can be modulated by site-directed mutagenesis of the heme binding pocket, and there is a direct correlation between the NO scavenging rates of the resulting mutant rHbs and vasoactivity in animal models [11]. 

Another approach to the amelioration of increased vasoactivity is to reduce extravasation of the cell-free Hb by increasing its size. Crosslinked polymeric Hbs exhibit reduced extravasation rates and appear to elicit an attenuated pressor response when administered to conscious rats and cats [12,13,14]. Thus, it appears that polymerized Hbs may have improved hemodynamic properties compared to the parent 64 kDa molecule. It is not clear whether an increase in hydrodynamic radius/molecular weight *per se*, or chemical modification of certain surface residues in Hb is responsible for this observed reduction in vasoactivity, since a 64 kDa rHb1.1 that was modified by treatment with glutaraldehyde (but not polymerized) also showed reduced vasoactivity in rats [12]. Nevertheless, polymeric Hbs are attractive targets in blood replacement therapy because they could be formulated as oxygen carrying therapeutics with equivalent oxygen binding capacity at lower oncotic pressures compared to a 64 kDa cell-free hemoglobin.

**Figure 1:**
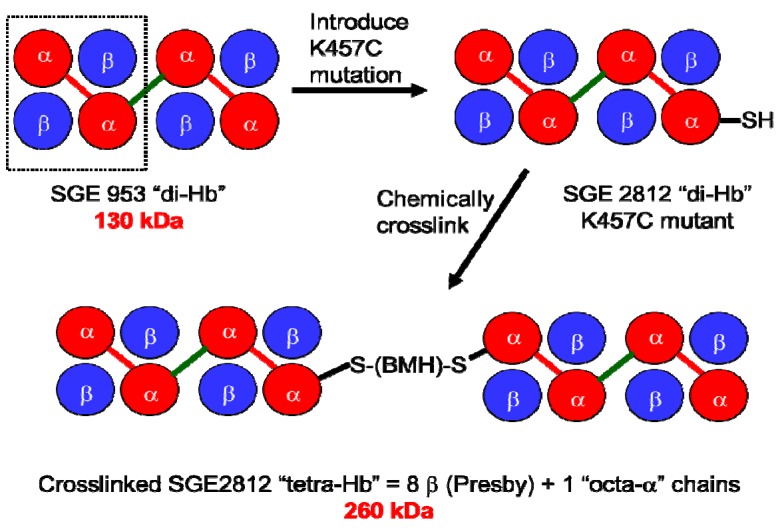
Covalent crosslinking of diα-globins (red) by gene fusion using a peptide linker (green) yields dihemoglobin (“di-Hb”). Introduction of a Cys residue into di-Hb, followed by chemical crosslinking with bismaleimidohexane (BMH), yields “tetra-Hb”. The subunit composition of rHb1.1 is shown within the dashed-line rectangle.

To address directly the effect of increased molecular size on the vasoactivity of rHb1.1, and to assess the feasibility of producing polymeric Hbs with defined oligomerization states, we produced a ~130 kDa “dihemoglobin”. “Dihemoglobin” as used herein refers to an oligomeric hemoglobin composed of four covalently linked α globin subunits which are noncovalently associated with four β globin subunits to yield a monodisperse 130 kDa protein complex (Figure 1). 

Given previous success with genetic fusion of α-globins [8], we pursued a strategy of generating tandem repeats of diα-globins as the basis for polymerization of the 64 kDa rHb1.1 molecules. A simple computer model suggested that a minimum of seven amino acids in an extended conformation would be required to link two rHb1.1 diα-globins without distortion of the terminal structures or overlap of the van der Waals surfaces . *A priori* a longer flexible linker might be best for minimizing effects on function (by imposing fewer steric constraints on the movement of the globin subunits during R- to T-state transitions), whereas a shorter linker might be better protected from proteolysis if the linker sequence proved to be a substrate for bacterial (or serum) proteases. Accordingly, dihemoglobins with 7aa (SGE 946) and 16aa (SGE 953) linkers were constructed to determine the effect of linker length on protein function and recovery.

The use of peptide linkers to create functional fusions of identical [4,15,16,17] or different [18,19,20,21] protein domains is well known, and a survey of linker regions listed in the Brookhaven protein database suggests that Thr, Ser, Gly and Ala are the most desirable constituent amino acids for a linker [22]. Thus, we opted for flexible linkers composed of Ser and Gly residues. One potential drawback to this simple approach is that larger polymers or aggregates might form as the result of misfolding of the tetra-α globin. At the outset of this study it was not known whether α globin sequences with extensions at *both* termini would fold correctly, or associate properly with the correct number of β-globins to yield a fully functional polymeric Hb.

To demonstrate that a 130 kDa polymeric hemoglobin with functional properties suitable for an oxygen carrying therapeutic can be produced solely by recombinant technology, we have produced and characterized two dihemoglobins, which differ only in the length of the linker between the second and third α globin subunits. To explore further the effects of increased molecular size on the vasoactivity of hemoglobin solutions, we also produced a Cys-containing dihemoglobin that can be chemically crosslinked to produce a monodisperse “tetrahemoglobin” of ~260 kDa (Figure 1). Both dihemoglobin and tetrahemoglobin show reduced vasoactivity compared to rHb1.1 in conscious rats.

## 2. Experimental Section

### 2.1. Gene Construction

**Figure 2:**
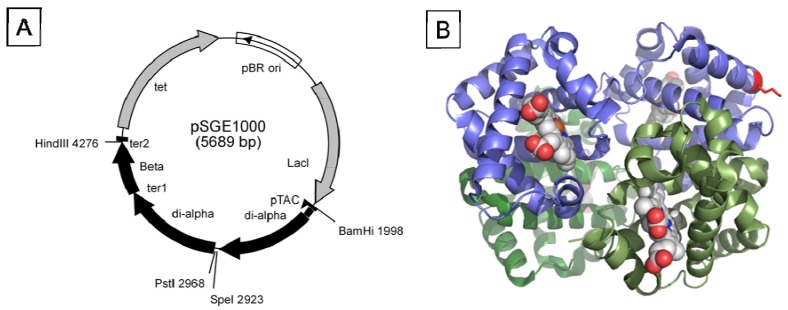
(**a**) Map of pSGE1000. The vector was designed to have unique SpeI and PstI sites flanking the fusion region. (**b**) X-ray crystal structure of deoxy-rHb1.1 (Reference [24]; PDB ID 1c7b), rendered using PyMol with the diα-globin in blue and K16 highlighted in red.

Oligonucleotides were synthesized on an Applied Biosystems DNA Synthesizer Model 392 using reagents supplied by the manufacturer. Restriction endonucleases were purchased from New England Biolabs. T4 DNA ligase was purchased from either New England Biolabs or Gibco-BRL. Plasmids pTZ19U and pBluescript II SK^+^ were purchased from BioRad and Stratagene, respectively. The expression plasmid pSGE705 was the source of the diα and β globin genes used to construct the dihemoglobins described herein. Plasmid pSGE705 encodes the diα and β globin genes of rHb1.1, and it has been described in detail elsewhere [23]. The rHb1.1 genes were cloned as a *BamHI/HindIII* DNA fragment into pTZ19U to yield a plasmid designated pTZ19U/705. Single-stranded DNA containing uracil substitutions was isolated and oligonucleotide-directed mutagenesis was performed using the Muta-gene Kit^®^ from BioRad according to the manufacturer's instructions. The first of two pTZ19U/705 clones was prepared using the oligonucleotide 5'-ACC GTT CTG ACT AGT AAA TAC CGT TAA TGA-3'. This oligonucleotide created a unique *SpeI* site at the 3' end of the diα gene, yielding plasmid pSGE1001. A second pTZ19U/705 clone was prepared using oligonucleotides 5'-GGA GGT TAA TTA ATG CTG TCT CCT GCA GAT-3' and 5'-CTG GTG GGT AAA GTT CTG GTT TGC GTT CTG-3'. The resulting clone (pSGE1002) incorporates a unique *PstI* site at the 5' end of the diα gene and deletes an *SpeI* site in the β gene compared to the parent genes. The assembly of the tetra-α gene construct was accomplished in four steps as follows: (1) removal of a diα gene cassette from pSGE1001 using *BamHI/SpeI* enzymes and gel purification of the DNA fragment, (2) pSGE1002 was cut with *PstI/BglII* enzymes to give a second diα gene cassette (which contains the 5' end of the β gene) and was also gel purified, (3) the following two oligonucleotides were annealed to make a dsDNA fragment which encodes the peptide linker SerGlyGlySerGlyGlySer flanked by 5' *SpeI* site and a 3' *PstI* site: 5'-CT AGT AAA TAC CGA TCG GGT GGC TCT GGC GGT TCT GTT CTG TCT CCT GCA-3' and 5'-GG AGA CAG AAC AGA ACC GCC AGA GCC ACC CGA TCG GTA TTT A-3', (4) the two diα fragments and the annealed oligonucleotide were ligated using T4 DNA ligase to create a tetra-α cassette with a 7 amino acid fusion peptide linking the two diα globins. This tetra-α cassette was then ligated as a *BamHI/BglII* fragment into pSGE705 from which the rHb1.1 genes had been removed as a *BamHI/BglII* fragment. The resulting tetra-α plasmid (pSGE1000, Figure 2) was transformed into *E. coli* strain SGE1661 [23] creating the expression strain SGE939. Due to the presence of four tandem repeats of the α globin sequence, it was not possible to sequence both coding and non-coding strands of the entire tetra-α gene by standard dideoxy methods. Single strand sequence of all four α globins was confirmed within pSGE1000. The linker region from pSGE1000 was sub-cloned as an *XhoI* fragment into pBluescript II SK^+^ and the expected sequence was obtained from both strands. pSGE705 is a low copy number plasmid, as is its daughter plasmid pSGE1000. A high copy number version of pSGE705, which contains the pUC *ori* is designated pSGE715. Another high copy number plasmid, pSGE720, differs from pSGE715 by a deletion of the plasmid copy of the *lacI* gene and the insertion of a polylinker [23]. The tetra-α and β genes from pSGE1000 were cloned into the pSGE720 background as a *BamHI/ HindIII* fragment. The resulting high copy number plasmid (pSGE1004) was transformed into *E. coli* strain SGE1675 [23] to make the expression strain SGE946. Another plasmid (pSGE1011) encoding the sixteen amino acid linker (SerGlyGly)5Ser between the diα globins was constructed by inserting a synthetic DNA cassette containing *SpeI* and *PstI* sticky ends into doubly digested pSGE1000. Plasmid pSGE1011 was transformed into SGE1661 to create expression strain SGE953. The tetra-α globin chain encoding the K16C (wild-type numbering) mutation in the C-terminal α-globin (amino acid 457 in the tetra-α globin chain) was generated by ligation of a normal diα-globin gene to a second diα-globin gene which contained the Cys mutation. The resulting K457C tetra-α globin chain was then subcloned into pSGE1011 and transformed into SGE1675 to produce the expression strain SGE2812.

### 2.2. Expression and Purification of Dihemoglobins

Fermentations were performed at 15 L or 100 L scale in vessels manufactured by Biolafitte at 30 ± 1 °C, controlling dissolved oxygen at 20% and glucose at 0–6 g/L. At OD_600_ 30 ± 2, cells were induced by adding isopropyl thiogalactoside (IPTG) to a final concentration of 100 µM, and the temperature of the fermentor was lowered to 28 ± 1 °C. Induction was carried out for 16 h. Hemin (50 mg/mL in 1 N NaOH) was added at 0, 3, 6, 9, and 12 h post-induction at a concentration of 0.07 g hemin/L of fermentor broth for each addition. After completion of the fermentation, cells were harvested by centrifugation and stored frozen at −80 °C. Frozen cells were partially thawed then chopped into small bits in a steel beaker using cold “break buffer” (40 mM Tris base, 1mM benzamidine) at a ratio of 2 mL break buffer per 1 gram of frozen cells. The diluted cells were resuspended by homogenization in a Waring Industrial Blender for 3–5 min on the low setting. The solution was allowed to settle for 5 min after homogenization and any foam was removed. Cells were lysed by a single passage of the homogenized cell solution through a Niro Panda™ cell disruption device (Niro Hudson) set at 800 bar. The lysate was collected on ice, sparged with CO gas, and the pH adjusted to pH 8.0 with sodium hydroxide. Sufficient Zn(OAc)_2_ was added to make the lysate 2 mM in Zn(OAc)_2_, and flocculating agent (Magnafloc 573-C, American Cyanamid) was added to 0.25% (v/v). The solution was then spun at 10,000 rpm in a JA-10 rotor at 4 °C for 60 min in a Beckman centrifuge. The red-colored supernatant was collected. All buffers were used at a temperature of 4 °C and were adjusted to the correct pH at 4 °C. Chelating Sepharose fast flow resin (Pharmacia) was charged with 2 to 3 column volumes (CV) of 20 mM Zn(OAc)_2_followed by 2 to 3 CV of 200 mM NaCl. The clarified lysate was loaded onto the column and washed with: 1 CV of 20 mM Tris•HCl, 50 mM NaCl pH 8.0; 2 CV of 20 mM Tris•HCl, 500 mM NaCl pH 8.0; 2 CV of 20 mM Tris•HCl, 50 mM NaCl pH 8.0; 10 CV of 10 mM imidazole, 50 mM NaCl pH 7.2; 4 CV of 20 mM sodium phosphate, 50 mM NaCl pH 6.5. The bound protein was then eluted with 20 mM Tris•HCl, 15 mM EDTA pH 8.0. The purified protein solution was concentrated to 20–40 mg/mL and buffer exchanged into 20 mM Tris•HCl, pH 8.8 using a Filtron Technology Corporation diafiltration apparatus equipped with 30 kDa MWCO membranes. The different size hemoglobins were separated by size exclusion chromatography (SEC) on S-200^®^ and S-300^®^ (GE Healthcare, Waukesha, WI, USA) columns linked in series. The columns were eluted using 10 mM phosphate pH 7.4, 150 mM NaCl (PBS) as the mobile phase. Appropriate fractions were pooled and buffer exchanged into 20 mM Tris•HCl pH 8.0 by diafiltration. The SEC-purified protein was bound to a thin bed Chelating Sepharose column charged with Zn(OAc)_2_, then reoxygenated by passing highly oxygenated 20 mM Tris•HCl pH 8.0 over the immobilized rHb for 7 h at 0 °C. Following reoxygenation, the protein was eluted with EDTA as described above and concentrated and diafiltered into 20 mM Tris•HCl pH 9.0. The reoxygenated SEC-purified protein was further purified by anion exchange chromatography on Q-Sepharose resin (GE Healthcare, Waukesha, WI, USA). The protein was loaded onto the column in 20 mM Tris•HCl pH 9.0. The column was washed with 3 CV 20 mM Tris•HCl pH 9.0 then eluted with 20 mM Bis-Tris pH 6.8. The purified proteins were formulated for preclinical studies in endotoxin-free PBS at final concentrations of 35–55 mg/mL. Tetrahemoglobin was produced from SGE2812 by treating the purified deoxygenated dihemoglobin at 150 mg/mL in 50 mM Tris pH 8.0 with a two-fold excess of bismaleimidohexane (Pierce, Inc., Salinas, CA, USA) for 2 h on ice. The crosslinked tetrahemoglobin was purified as described above using S-200 and S-300 SEC, followed by Q-Sepharose to remove endotoxins prior to formulation for preclinical testing.

### 2.3. Determinations of Apparent Molecular Weight

The globin chains of the purified material were separated by C4 reversed phase high performance liquid chromatography (RP-HPLC) on a Hewlett Packard model 1090 HPLC equipped with a Vydac 5 µm 0.46 × 25 cm C4 column using a gradient of acetonitrile (ACN) in water (both containing 0.1% trifluoroacetic acid) as the mobile phase with a flow rate of 1.0 mL/min. The 75 min gradient elution was established as follows: 3 min at 30% ACN, a linear gradient from 30% ACN to 37% ACN over 12 min, a linear gradient from 37% ACN to 50% ACN over 60 min. The separated globins were analyzed by positive ion electrospray mass spectroscopy (Vestec model 201 Thermospray LC-MS). Analytical SEC was performed on a Hewlett Packard model 1090 HPLC equipped with Superose-12^®^ and Superose-6^®^ columns (GE Healthcare) linked in series using PBS as the mobile phase with a flow rate of 0.5 mL/min. Apparent molecular weights were calculated from retention times after calibrating the columns with protein MW standards (Sigma Chemical Co., St. Louis, MO, USA). Samples for analytical ultracentrifugation were prepared in PBS buffer and loaded at three different concentrations (sample/buffer ratios were 1:1, 1:2 and 1:3). The samples were loaded into a six sector cell, 3 samples per cell with associated PBS buffer blank. The cells were loaded into a 4 hole An-60 Ti titanium rotor which was then subsequently spun at 9,000, 12,000 and 15,000 rpm in a Beckman analytical ultracentrifuge model XL-A. Protein absorbance was monitored using a Xenon flash lamp light source at 540 nm. Best fit curves to the data (absorbance versus radial position) were generated using Origin v3.78 and the associated software provided by Beckman for data analysis. The apparent particle sizes of purified di- and tetrahemoglobins were determined by dynamic light scattering using a NICOMP 370 HPL submicron particle sizer as described elsewhere [12].

### 2.4. Determination of in vitro Functional Properties of Dihemoglobins

The oxygen affinity and cooperativity of the purified protein were determined at 37 °C in 50 mM HEPES (pH 7.4) 100 mM NaCl using a Hemox analyzer (TCS Medical Products). Data were analyzed as described elsewhere [25] and the values of P_50_ and n_max_ reported are calculated from curve fitting. The stoichiometry of ligand binding was determined by titrating deoxy dihemoglobin with CO-saturated buffer at 20 °C. Deoxy dihemoglobin at 1.45 µM in PBS (theoretically 11.7 µM in heme sites) was reduced by addition of sodium dithionite to give a final concentration of 1 mM Na_2_S_2_O_6_. At 635 torr and 20 °C CO-saturated buffer is calculated to be 870 µM in CO [26]. Over the course of the titration, 5 µL aliquots of CO-saturated PBS were added to 4 mL of the reduced deoxy dihemoglobin, mixed for 3 min and then a UV/VIS absorbance spectrum was recorded (Hewlett Packard Model 8452A Diode Array UV/Vis) until further addition of CO-saturated buffer produced no change in absorbance at 418 nm. Volume percent of O_2_ was determined using a LEXO_2_CON-K (Lexington Instruments Corp., Lexington, KY, USA) calibrated with standard injections of air. Solutions of purified dihemoglobin in PBS were compared to a PBS blank. The percent of O_2_ was recorded as the average of five determinations. For these calculations the concentrations of dihemoglobin solutions were measured by converting the dihemoglobins to the cyanomet form and measuring absorbance at 540 nm. The oxy/deoxy difference spectrum [27] of dihemoglobin between 450–700 nm was generated by subtracting the deoxy-diHb spectrum from the oxy-diHb spectrum. NO scavenging rates for dihemoglobins were determined as previously described [10].

### 2.5. Determination of Circulating Halflife, Hemodynamic Response and Extravasation in Vivo

Circulating halflife was determined in male Sprague-Dawley rats that had been chronically instrumented with venous catheters 4–6 days before experimentation. Top-load doses of 350 mg/kg of protein were administered via intravenous infusion at a rate of 0.5 mL/min to six rats each in experimental (dihemoglobin) and control (rHb1.1) groups. Blood (0.3 mL) was collected from the tail vein into heparanized tubes and centrifuged to obtain plasma at 0, 0.5, 1, 2, 4, 8, 12 and 24 h post infusion. Plasma rHb concentration was determined by converting rHb to the cyanomet form and measuring the absorbance at 540 nm (extinction coefficients were determined independently by iron analysis). Time-dependent curves of plasma hemoglobin concentrations were fitted to data using single exponential equations. Peritoneal lavage was used as a second method for determination of circulating halflife. rHb1.1 or dihemoglobin was administered to separate groups of conscious Balb/C mice via tail vein injection (500 mg/kg). At 1 h or 3 h post-administration the mice were euthanized by CO_2_ asphyxiation and 8 mL of PBS were injected into the peritoneal cavity. Peritoneal lavage samples (1–6 mL) were withdrawn and centrifuged to remove cellular debris. Hemoglobin concentrations in the peritoneal fluid were subsequently determined by an ELISA-based assay. Hemodynamic responses to hemoglobin administration were obtained in conscious, unrestrained rats. Male Sprague-Dawley rats were chronically instrumented with indwelling arterial and venous catheters at least 48 hours prior to experimentation. Top-load doses of 350 mg/kg of rHb1.1, dihemoglobin, tetrahemoglobin, or human serum albumin (HSA, 50 mg/mL) were administered to separate groups of rats (n = 6 in all groups) via intravenous infusion at a rate of 0.5 mL/min. Arterial pressure was monitored continuously for 30 min prior to and 90 min following hemoglobin administration.

## 3. Results and Discussion

**Figure 3:**
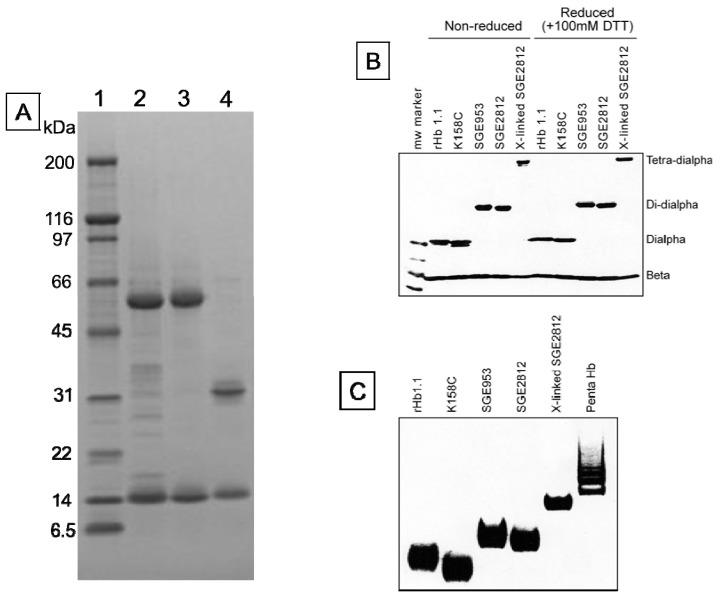
Tris-glycine gels (4%–20%) stained with coomassie brilliant blue. (**a**) SDS-PAGE of SGE 953 and rHb1.1. Lane 1, MW markers. MWs in kDa are indicated. Lane 2, partially purified dihemoglobin from SGE 953. This material is the collected fraction from the first chromatography step. Lane 3, fully purified dihemoglobin from SGE 953. Lane 4, purified rHb1.1; (**b**) SDS-PAGE of rHb1.1, rHb1.1 K158C, SGE 953 and SGE 2812 before and after chemical crosslinking; (**c**) Native PAGE of rHb1.1, rHb1.1 K158C, SGE 953, SGE 2812 before and after chemical crosslinking, and a glutaraldehyde-crosslinked “penta-Hb”.

Polymeric hemoglobins have potential advantages over 64 kDa hemoglobins as cell-free injectable therapeutics because larger MW hemoglobins could be formulated as oxygen carrying therapeutics with equivalent (or greater) oxygen binding capacities at lower oncotic pressures compared to a 64 kDa cell-free hemoglobin [1,3]. Initially, Somatogen approached the generation of a polymeric hemoglobin solution via chemical crosslinking of rHb1.1 with glutaraldehyde [12]. This was undertaken to determine the effects of increased molecular size on vasoactivity, as others had reported minimal vasoactivity with crosslinked Hbs [28]. Solutions of chemically crosslinked Hbs contain multiple polymer species (see Figure 3). To better define the relationship between polymer size and vasoactivity, we sought to take advantage of the power of recombinant DNA technology to generate polymeric rHbs of defined molecular weight.

Genes for three different dihemoglobins have been constructed and cloned into low and high copy expression vectors (Figure 1 and Figure 2). The design of the expression vector allows for facile insertion of a linker of any desired amino acid sequence between the unique *SpeI* and *PstI* restriction sites flanking the fusion region. At the end of fermentation there is no evidence from restriction mapping of other plasmid species which might arise from the deletion of α globin sequences (data not shown), thus the plasmid appears to be stable over many generations.

**Figure 4:**
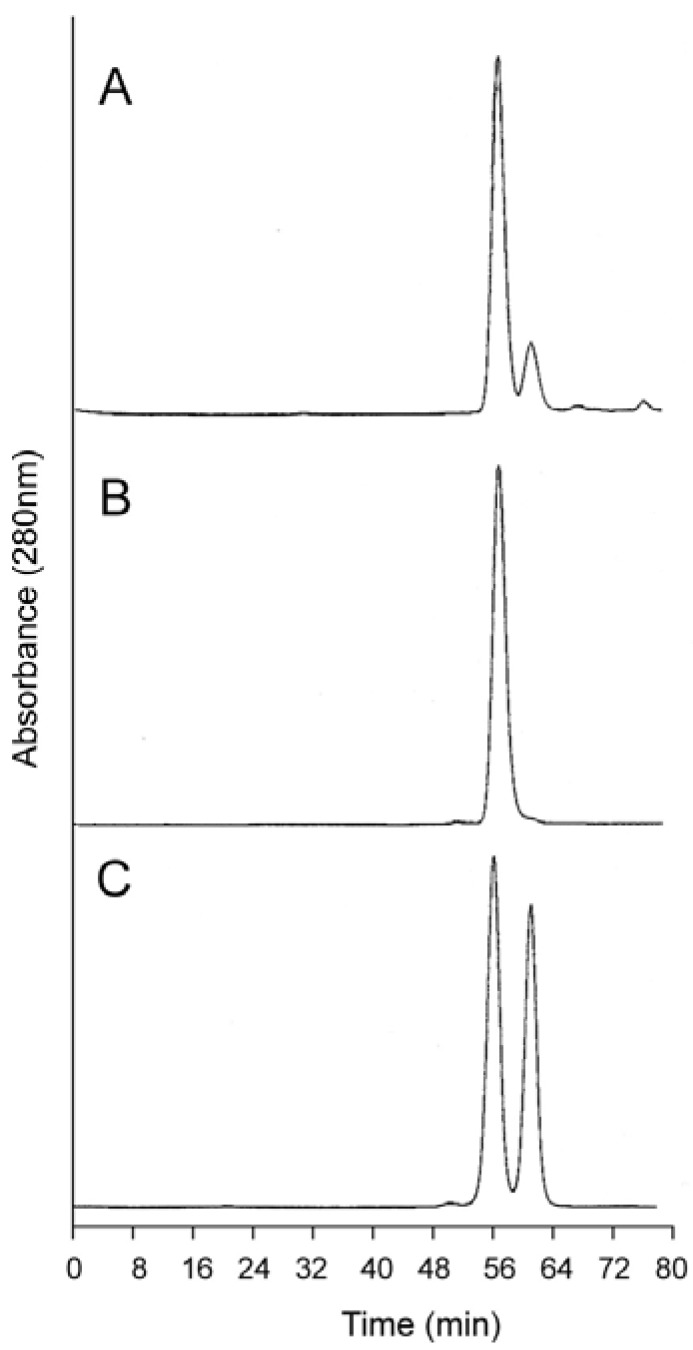
Analytical size exclusion chromatography of dihemoglobin SGE 953. (**a**) Partially purified SGE 953. This material is the collected fraction from the first chromatography step (see lane 2 of Figure 3A); (**b**) Purified SGE 953; (**c**) Coinjection of purified SGE 953 and purified rHb1.1.

We were concerned that the approach to the generation of larger hemoglobins described herein would yield unstable and/or misfolded proteins. Dihemoglobin is expressed at high levels in *E. coli* and purification of dihemoglobins from bacterial lysate to greater than 95% homogeneity (Figure 3) is readily achieved using the three column process described in *Experimental Procedures*. After the final purification step the major contaminants are monohemoglobin species (Figure 4B). Figure 4A shows an analytical SEC chromatogram of the dihemoglobin protein recovered from a fermentation of expression strain SGE 946 after the first column purification. At this stage the protein mixture is 82% dihemoglobin and 18% monohemoglobin. For all the dihemoglobin preparations described in this report, 10%–20% of the globins recovered after the first column purification appear to be monohemoglobin species. Mass spectral and sequencing analyses of the monohemoglobin fraction indicate that it is heterogeneous and suggest a non-specific degradation of the dihemoglobin molecule. Misfolded proteins are likely to be degraded, however, correctly folded dihemoglobins may also be substrates for degradative processes. It is possible that the presence of the peptide linking the diα globins causes local destabilization of the hemoglobin in the fusion region, thereby increasing the susceptibility of this region to proteolysis.

The proper assembly of the desired tetra-α globin and four β-globin chains was confirmed by SDS-PAGE (Figure 3) and mass spectral analysis of the isolated globin chains. Integration of the area under the peaks of analytical reversed phase HPLC chromatograms indicates that the tetra-α and β globins are present in the expected stoichiometry of 1:4 (data not shown).

Analytical SEC shows good separation between rHb1.1 and the faster migrating dihemoglobin when the two proteins are co-injected (Figure 4C). The apparent molecular weights of the purified dihemoglobins were estimated from their retention times on analytical SEC columns that had been calibrated with protein MW standards (Table 1). These analyses yielded MWs for the dihemoglobins that are twice that for rHb1.1. Dynamic light scattering (Table 1) also shows that particle sizes of the hemoglobins increase going from rHb1.1 (64 kDa) to SGE 946 or 953 (130 kDa) to SGE 2812 (260 kDa).

**Table 1:** Apparent molecular weights and particle sizes of dihemoglobins.

Hemoglobin	Calculated MW (kDa)	Estimated MW from SEC (kDa)	Observed MW from sedimentation (kDa)	Observed diameter from light scattering (nm)
rHb1.1	64.6	52.5	63–64	6.0
SGE 946	130	102	126–128	10.4
SGE 953	130	102	127–129	9.7
SGE 2812	260	173	n.d.	12.4

A quantitative determination of MW was made by analytical ultracentrifugation. Observed values of MW obtained from curve fitting are in excellent agreement with expected MWs for the three dihemoglobins and rHb1.1 (Table 1). By varying the concentration of the protein and analyzing the signal at various speeds, the individual curves could be combined and fit globally, thus reducing the influence of either concentration or rotor speed. The curve fitting equation included a term for non-ideality that arises due to the finite size of the protein and its associated charge; both can lead to an apparent decrease in MW with increasing concentration [29]. The samples displayed homogeneity, as no increase in the molecular weight from the meniscus position to the bottom of the cell was detected and plots of ln(conc) *vs.* r^2 ^did not exhibit upward curvature.

The suitability of dihemoglobin to function as an O_2_ delivery platform was assessed by measuring equilibrium oxygen binding, oxygen content, and the stoichiometry of ligand binding. Dihemoglobin displays equilibrium oxygen binding behavior similar to rHb1.1 (Table 2), with observed values of P_50_ and n_max_ that are suitable for an oxygen-delivering therapeutic. The dihemoglobins showed a modest reduction in the values of P_50_ and n_max_ relative to rHb1.1. The observed values of these parameters indicate that dihemoglobin could function as an efficacious oxygen delivery molecule. Theoretical fractional saturation of hemoglobin is calculated using the following equation:

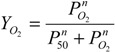

where 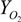 is fractional saturation, 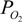 is the partial pressure of oxygen, *P_50_* is the 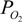 required to achieve 50% saturation of the ligand binding sites, and n is the Hill coefficient. Given the observed values of these constants for the dihemoglobins (Table 2) and assuming an oxygen partial pressure of 90 torr in the lungs and 40 torr in the capillary beds, these molecules could theoretically release 21% of bound oxygen compared to a theoretical 30% release of oxygen from whole blood.

**Table 2:** Oxygen binding and NO scavenging rates for dihemoglobins.

	rHb1.1	SGE 946	SGE 953
P_50_ (torr)	31	24	25
n_max_	2.2	2.0	2.1
*k*’_NO_ (μM^−1^s^−1^)	71	71	66

The observed reductions in P_50_ and n_max_ may reflect stabilization of the R state conformation that results from the linking of the two hemoglobins. Marked reductions of P_50_ and n_max_ have been reported for natural [30] and directed mutants [31] of the penultimate Tyr in α globin (Tyr140-α). This residue is thought to stabilize the T-state structure and promote tetramer assembly [31]. The linker may alter the conformation of this residue in dihemoglobin and thereby alter the equilibrium oxygen binding properties of the dihemoglobin relative to rHb1.1. The covalent linkage of the tetramers may also result in inter-tetramer interactions that perturb the T-R equilibrium in favor of the R-state.

**Figure 5:**
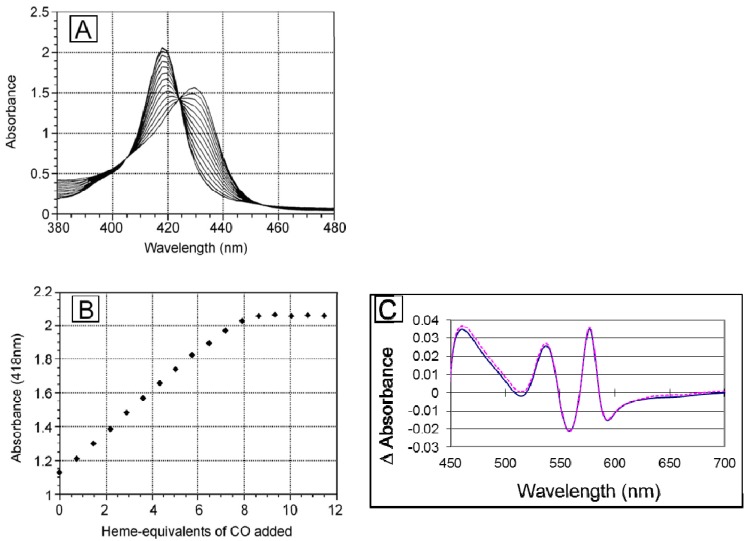
Ligand binding to SGE 953. (**a**) The stoichiometry of ligand binding was determined by titrating deoxy dihemoglobin with CO-saturated buffer at 20 °C. An overlay of the spectra recorded after each CO addition during the titration is shown; (**b**) The data from panel (a) are plotted at 418 nm; (**c**) The (oxy di-Hb)-(deoxy di-Hb) difference spectra for SGE 953 (magenta, dashed line) and rHb1.1 (blue, solid line).

To ascertain that the linker does not render any of the ligand binding sites non-functional, we titrated the deoxy form of the dihemoglobin with CO-saturated buffer (Figure 5). Dihemoglobin has 7.9 (of 8 expected) CO binding sites (Figure 5), and rHb1.1 has 3.7 (of 4 expected) CO binding sites (data not shown). When the spectra acquired during the titration are overlaid (Figure 5A), three isosbestic points emerge, indicating that there are not multiple absorbing species in solution and that the heme environment is similar at all ligand binding sites in the dihemoglobin molecule. In addition, the O_2 _content of dihemoglobin and tetrahemoglobin prepared for preclinical studies was measured as described in *Experimental Procedures* and shown to be 95% of the expected value (data not shown). Finally, the oxy-deoxy difference spectra for rHb1.1 and dihemoglobin (Figure 5C) are essentially identical, indicating no significant perturbation of the heme binding pocket in dihemoglobin compared to rHb1.1. These data taken together support the conclusion that covalent linkage of the hemoglobins has not hampered ligand binding at any of the eight heme centers in the dihemoglobin molecule.

Given the encouraging *in vitro* characteristics of dihemoglobin we undertook further studies to compare the *in vivo* properties of these novel hemoglobin polymers to the well-characterized recombinant hemoglobin rHb1.1. Administration of hemoglobin solutions has resulted in undesired vasoactivity in humans [2,3,4]. Two different mechanisms have been proposed to account for this vasoactivity. One attributes the effects to increased O_2_ concentrations in non-capillary vasculature, via facilitated diffusion of O_2_ by cell-free hemoglobin (see [32] and references therein). The other proposes that oxy-Hb extravasation and the subsequent scavenging of NO by oxy-Hb is responsible for the observed effects [10]. Polymeric hemoglobins have been reported to possess increased retention in circulation [13,14], and we hypothesized that a larger molecule, such as dihemoglobin, would extravasate to a lesser extent than rHb1.1. Attenuated extravasation would presumably reduce or eliminate NO scavenging by oxy-Hb and thereby lead to a reduced pressor response. The rates of NO scavenging of oxydihemoglobin and oxy-rHb1.1 are identical (Table 2), thus the effect of apparent MW on the reduction of the pressor response *in vivo* can be directly compared for rHb1.1 and dihemoglobin.

**Figure 6:**
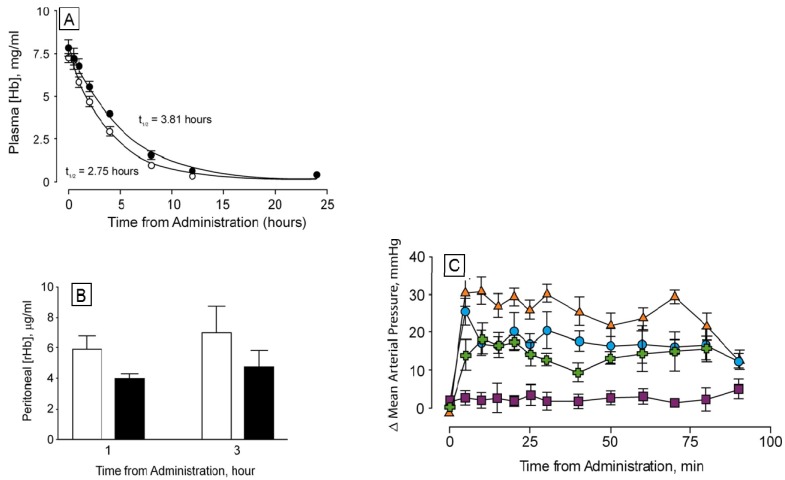
Determination of circulating halflife and hemodynamic response of di-Hb *in vivo* (**a**) Circulating halflife was determined in male Sprague-Dawley rats. Top-load doses of protein were administered via intravenous infusion to six rats each in experimental (SGE 953, filled circles) and control (rHb1.1, open circles) groups. Time-dependent curves of plasma hemoglobin concentrations are shown; (**b**) Hemoglobin concentrations in peritoneal lavage fluid at two time points following administration of rHb1.1 (open bars) or dihemoglobin from SGE 953 (filled bars) to separate groups of Balb/C mice; (**c**) Changes in mean arterial pressure following administration of hemoglobins to conscious, unrestrained rats. Top-load doses of rHb1.1 (filled triangles, orange), SGE 953 di-Hb (filled circles, blue), crosslinked SGE 2812 tetra-Hb (filled crosses, green), or HSA (50 mg/mL, filled squares, purple) were administered to separate groups of rats (n = 6 in all groups).

Pharmacokinetic measurements show that the circulating halflife of dihemoglobin is greater than that of rHb1.1 by a factor of 1.4. When administered at a dose of 350 mg/kg, rHb1.1 has a halflife of 2.75 h whereas the halflife of dihemoglobin from SGE 953 is 3.81 h (Figure 6A). Dihemoglobin also extravasates to a lesser degree than rHb1.1 (Figure 6B). Peritoneal lavage after hemoglobin administration in mice showed approximately 30% less dihemoglobin had entered the peritoneal cavity compared to rHb1.1. The difference between rHb1.1 and dihemoglobin was statistically significant at the 1 h time point (p < 0.05). In addition to increased retention in the vasculature, dihemoglobin does not raise mean arterial pressure (MAP) to the same extent as rHb1.1 when administered to conscious rats (Figure 6C). The difference between rHb1.1 and dihemoglobin was statistically significant (p < 0.05, repeated measures ANOVA). Data obtained in experiments with a related dihemoglobin show that most of the MAP elevation can be attributed to an increase in cardiac output rather than peripheral vasoconstriction (M. Doyle, unpublished). Taken together, these data support the hypothesis that increased vascular retention of hemoglobin can minimize the observed pressor response, and that even a relatively modest two-fold increase in MW can increase vascular retention.

**Figure 7:**
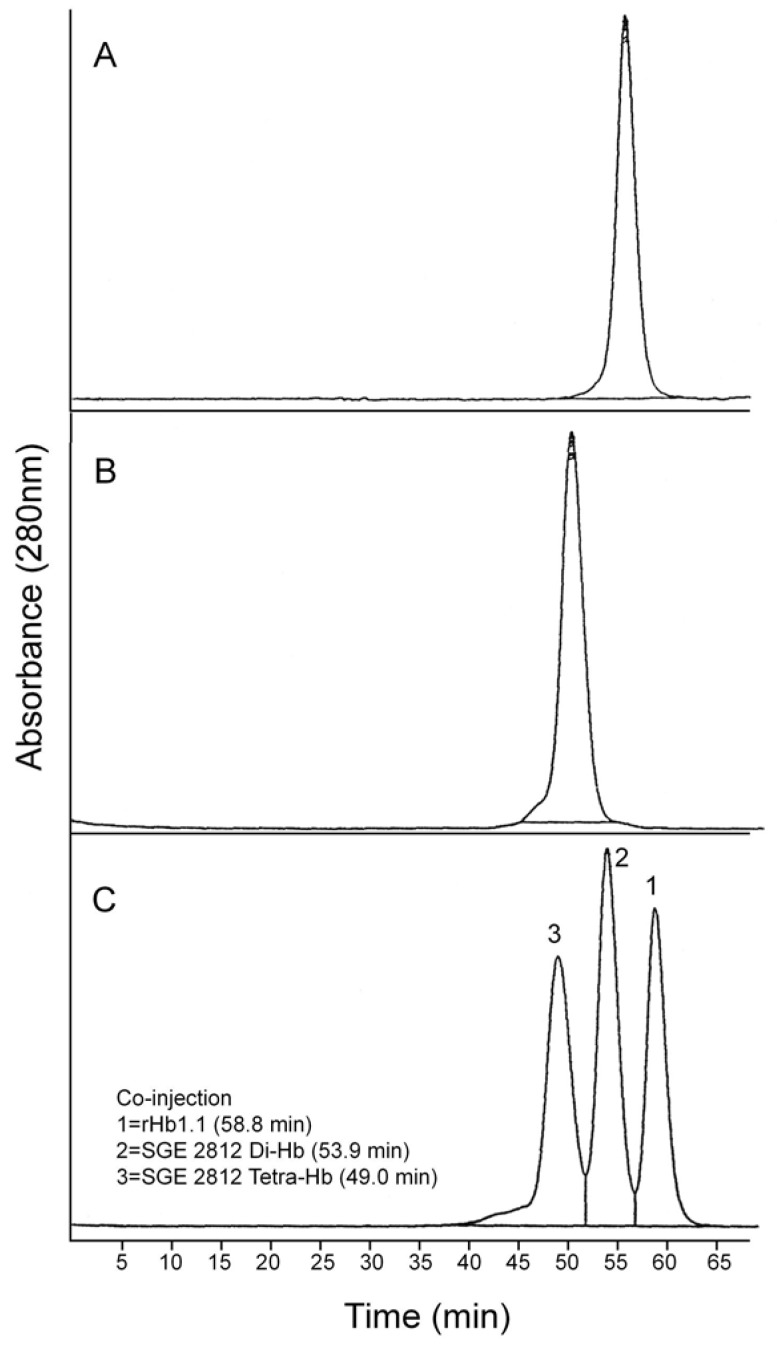
Analytical size exclusion chromatography of dihemoglobin SGE 2812. (**A**) Purified SGE 2812 prior to crosslinking; (**B**) Purified SGE 2812 after crosslinking with bismaleimidohexane; (**C**) Coinjection of rHb1.1 (t_r_ = 58.8 min) and purified SGE 2812 before (t_r_ = 53.9 min) and after (t_r_ = 49.0 min) BMH crosslinking.

To explore further the effect of increased MW on vasoactivity, we produced a 240 kDa tetrahemoglobin by chemical crosslinking of a dihemoglobin containing a single reactive Cys residue in the C-terminal α-globin of the tetra-α-globin chain (Figure 1). Lysine 16 (wild-type Hb numbering) is known to react with chemical modifying agents such as glutaraldehyde (12) and is solvent accessible in the rHb1.1 structure (Figure 2B). We mutated this site (amino acid residue 457 in SGE 953) to produce a di-Hb with a uniquely reactive site for chemical crosslinking under deoxy conditions (under oxy conditions β-Cys93 is also reactive toward maleimide reagents). This combined site-directed mutagenesis and chemical crosslinking approach allowed us to produce a monodisperse tetra-Hb (Figure 7B). A preparation of glutaraldehyde linked “penta-Hb” contains significant concentrations of tetra-, hexa- and septa-Hb polymers in addition to the predominant penta-Hb (see Figure 3C). In contrast, SGE 2812 is composed of a single polymeric species (Figure 3B,C). 

The tetrahemoglobin produced in this fashion shows reduced elevation in MAP compared to rHb1.1, but the observed change is not significantly different from that seen for dihemoglobin (Figure 6C). This observation is consistent with a recent report that glutaraldehyde crosslinked poly-Hb solutions containing oligomers of <500 kDa exhibited significant vasoactivity, whereas those containing oligomers of >500 kDa showed minimal vasoactivity in preclinical testing [33].

## 4. Conclusions

We have demonstrated that a 130 kDa human hemoglobin polymer can be produced in bacteria and purified to greater than 95% homogeneity. Furthermore, the dihemoglobin retains ligand binding function with values for P_50_ and n_max_ that are suitable for an O_2_ carrying therapeutic. In addition, dihemoglobin exhibits longer vascular retention and reduced vasoactivity compared to a 64 kDa hemoglobin. This technology provides a molecular scaffold for the protein engineering of polymeric hemoglobin solutions derived entirely from a recombinant source and/or other hemoglobin-based therapeutics with functional properties tailored to specific clinical applications [34]. 
